# Evaluation of plant-based antimicrobials in controlling antimicrobial resistance gene spread in poultry production continuum

**DOI:** 10.3389/fvets.2026.1837338

**Published:** 2026-07-28

**Authors:** Poonam Vinayamohan, Leya Susan Viju, Abraham Joseph Pellissery, Meera Surendran Nair, Annie M. Donoghue, Kumar Venkitanarayanan

**Affiliations:** 1 Department of Animal Science, University of Connecticut, Storrs, CT, United States; 2 Department of Comparative, Diagnostic & Population Medicine, University of Florida, Gainesville, FL, United States; 3 Department of Veterinary and Biomedical Sciences, Pennsylvania State University, State University, PA, United States; 4 Poultry Production and Product and Safety Research, ARS, USDA, Fayetteville, AR, United States

**Keywords:** antimicrobial resistance, horizontal gene transfer, phytochemicals, plasmid, *Salmonella*

## Abstract

Antimicrobial resistance poses a looming global health challenge impacting human, animal, and environmental health sectors, with horizontal gene transfer (HGT) playing a critical role in the dissemination of resistance genes among bacteria. To address this challenge effectively, a comprehensive strategy encompassing animal health and environmental management is essential. Therefore, this study aims to investigate the efficacy of phytochemicals on the conjugative transfer of an antimicrobial resistance gene, *bla*_TEM,_ between multidrug-resistant *Salmonella* Heidelberg, a major food-borne pathogen in poultry and commensal *Escherichia coli*. The effect of four phytochemicals, namely trans-cinnamaldehyde (TC), carvacrol (CR), beta-resorcylic acid (BR), and caprylic acid (CA) were determined across diverse environments, including bacteriological broth, chicken manure, and water. Further, the efficacy of in-feed supplementation of TC, CR and TC-CR combinations in reducing HGT frequency in broiler chicken ceca *ex vivo* was also studied. The HGT frequency was calculated as the ratio of the number of transconjugant CFU per mL to the number of recipient CFU per mL. Exposure to CR and BR reduced transconjugant counts and HGT frequency in broth and chicken manure, while CR and CA showed similar effects in water (*p <* 0.05). Additionally, in-feed supplementation 0.5% TC decreased HGT frequency in chicken ceca *ex vivo* (*p <* 0.05). There was also a significant reduction in the transcription of conjugation genes in *Salmonella* treated with phytochemicals (*p <* 0.05). These findings suggest the potential of phytochemicals to mitigate the spread of *bla*_TEM_ in the poultry production continuum, although additional studies in a large number of samples, including chickens, are imperative to validate these results.

## 1 Introduction

Despite continued efforts to reduce antibiotic usage, antimicrobial resistant (AR) infections are on the rise, causing substantial morbidity, mortality and economic costs (1–5). The transfer of antimicrobial resistance traits amongst bacterial populations, predominantly via horizontal gene transfer (HGT), particularly conjugation, has been a critical factor in the proliferation of AR bacteria (6–8, 82). The livestock gastrointestinal tract serves as a critical reservoir for antimicrobial resistance by driving high rates of plasmid-mediated conjugation within its dense microbial ecosystem. This specialized microenvironment accelerates horizontal gene transfer by promoting sustained bacterial colonization, biofilm development, and continuous exposure to selective pressures from agricultural antibiotic use (9, 10).

The improper and widespread use of antibiotics in food animals can potentially facilitate the propagation of AR bacteria, including *Salmonella*, into the farm environment via animal excreta (9, 11, 12). This in turn contaminates various environmental elements, including animal gut, soil, sewage, treated drinking water, river water, and the air surrounding farm environments, thus heightening the risk of AR bacteria transmission to humans through the food chain or direct contact (13–18). *Salmonella* Heidelberg, a major foodborne disease agent frequently found in poultry products, showcases resistance to *β*-lactam antibiotics, a globally prescribed antibiotic class (19, 20). The application of manure from antibiotic-treated animals to soil, which may contain AR genes, can potentially lead to the introduction of these resistance traits into the environment. This, in turn, can impact the soil resistome and potentially enter the food chain, posing risks to human health (17, 21–23). For instance, the plasmid-mediated gene *bla*_TEM-1_ serves as a significant marker of resistance that utilizes mobile genetic elements, such as transposons, to undergo HGT to other environmental bacteria, including commensals like *Escherichia coli* (19). It has been recorded that AR bacteria may exist in manure from animals without a history of antibiotic use, signifying the natural occurrence of bacteria inherently resistant to antibiotics within the animal gastrointestinal system (24–26). Therefore, to reduce the prevalence and spread of AR in food animal production, alternative strategies in addition to prudent antibiotic usage needs to be implemented.

Phytochemicals, with their multifaceted antimicrobial mechanisms, are being increasingly proposed as potential alternatives to antibiotics in controlling bacterial resistance (27–33). Phytochemicals belonging to different chemical classes such as aldehydes, phenols, and fatty acids exhibit distinct modes of action against bacterial structures which may uniquely interfere with the cellular machinery involved in conjugation. For instance, trans-cinnamaldehyde acts as a potent quorum-sensing inhibitor that downregulates conjugation-related genes, suppresses biofilm formation, and disrupts bacterial membranes (34, 35). Similarly, the plant-derived phenol carvacrol induces rapid cell membrane damage (36), a mechanism that can physically compromise conjugative plasmid transfer. *β*-resorcylic acid serves as a stable, economically viable organic acid that remains active in complex matrices such as cecal contents; it functions as a non-dissociated weak acid to modulate the local environment and downregulate critical bacterial virulence genes (37). Caprylic acid induces antimicrobial activity by altering plasma membrane permeability through surfactant action and causing intracellular acidification via passive diffusion (38).

In addition, caprylic acid and trans-cinnamaldehyde have shown antimicrobial activity against Salmonella in our previous studies (39, 83). Phytochemicals have shown promise in reducing bacterial pathogen loads in poultry gut as well as in environments such as manure, poultry drinking water, and soil (28, 40, 41), but research into their ability to inhibit HGT is limited.

The objective of this study was to investigate the efficacy of four plant-derived compounds, namely trans-cinnamaldehyde, carvacrol, *β*-resorcylic acid, and caprylic acid, in inhibiting the horizontal gene transfer (HGT) of antibiotic resistance from multidrug-resistant *Salmonella* Heidelberg to commensal *Escherichia coli*. Specifically, we investigated the suppression of conjugative IncI1_Alpha plasmid transfer carrying the *bla*_TEM-1B_ gene by these phytochemicals, while elucidating the underlying cellular mechanisms driving this inhibition. We hypothesized that these phytochemicals would significantly reduce conjugation frequency at sub-inhibitory concentrations by potentially downregulating key plasmid transfer genes required for conjugal bridge formation.

## 2 Materials and methods

### 2.1 Bacterial strains and culture conditions

Multidrug-resistant *S*. Heidelberg (V6FA: resistant to ampicillin, streptomycin, tetracycline, sulfamethoxazole, chloramphenicol, and trimethoprim-sulfamethoxazole) was used as the donor strain, and an antibiotic-susceptible strain of non-pathogenic *E. coli* OP50 was used as the recipient strain. The bacteria were cultured in Luria-Bertani broth (LB) by incubation at 37 °C for 24 h. *S.* Heidelberg was streaked on Xylose Lysine Deoxycholate (XLD, Difco BBL) agar plates containing 100 μg/mL of ampicillin (Sigma-Aldrich, St. Louis, MO) to confirm resistance towards ampicillin. Additionally, *Salmonella* cultures were streaked on XLD plates containing nalidixic acid to confirm their susceptibility towards nalidixic acid (Sigma-Aldrich, St. Louis, MO).

### 2.2 Preparation of nalidixic acid resistant *Escherichia coli* mutants

For selective plating of the recipient bacterium in the conjugation experiments, *E. coli* OP50 were rendered resistant to 50 μg/mL of nalidixic acid, as described by Blackburn and Davies (42). Freshly grown *E. coli* OP50 was inoculated into LB broth containing 5 μg/mL nalidixic acid and incubated at 37 °C for 24 h. After incubation, the culture was streaked on LB agar containing 5 μg/mL nalidixic acid and similarly incubated. Colonies from the resulting incubation were subsequently inoculated into LB broth containing double the concentration of nalidixic acid (10 μg/mL). Subsequent subcultures were repeated as previously outlined with stepwise increments of nalidixic acid until growth in 50 μg/mL of nalidixic acid was established. The *E. coli* count was determined by plating 100 μL of ten-fold serial dilutions of the culture on duplicate Tryptic Soy Agar (TSA) and Sorbitol Mac Conkey agar (Sigma Aldrich, Boston, MO) containing 50 μg/mL of nalidixic acid, followed by incubation at 37 °C for 24 h.

### 2.3 Determination of sub-inhibitory concentration of phytochemicals in LB broth, manure and water

The four phytochemicals with ≥ 98% purity were purchased from Sigma Aldrich, St. Louis, MO, and were dissolved in dimethyl sulfoxide (DMSO; Sigma Aldrich, Boston, MA) to obtain a 10% stock solution. The sub-inhibitory concentration (SIC) is defined as the highest concentration of a compound that did not inhibit bacterial growth after 24 h of incubation (43, 44). The SIC of TC, CR, CA, and BR against the donor and recipient bacterium was determined in LB broth, manure, and water by the agar plating method, as previously described (43, 44). Tubes containing LB broth (10 mL) and 1 to 10 μL of each phytochemical in increments of 0.5 μL were inoculated separately with *Salmonella* or *E. coli* at ~ 6 log_10_ CFU/mL and incubated at 37 °C for 24 h. Tubes without any added phytochemical were included as controls in addition to DMSO and pH controls. After incubation, the samples were serially diluted (1:10) in PBS, plated on TSA, and incubated at 37 °C for 24 h before counting the colonies. For the manure experiments, tubes containing 2.5 g of autoclaved manure (in 10 mL maximum recovery diluent, MRD; Oxoid, Cheshire, England) (45) containing 1 to 150 μL of each phytochemical in increments of 5 μL were inoculated separately with Salmonella or *E. coli* at ~ 8 log_10_ CFU/mL and incubated at 25 °C for 24 h. While autoclaving eliminates background microbiota to allow precise tracking of donor and recipient populations, it is acknowledged that this sterilization alters the baseline microbial competition and ecological interactions present under actual field conditions. Similarly, experiments were done in water (10 mL) containing 1 to 20 μL of each phytochemical in increments of 0.5 μL and inoculated separately with *Salmonella* or *E. coli* at ~ 6 log_10_ CFU/mL and incubated at 25 °C for 24 h. The experiment was done in duplicate and repeated six times.

### 2.4 Conjugation experiments in broth

Conjugation experiments were conducted in LB broth, as previously mentioned (46). Ampicillin resistant *S*. Heidelberg (donor) and nalidixic acid resistant *E. coli* (recipient) were incubated separately in 30 mL of LB broth at 37 °C for 18 h to form stationary-phase cultures.

containing approximately 8 log_10_ CFU/mL. A 1-mL aliquot from each stationary-phase culture was serially diluted twice in 9-mL volumes of LB broth to form inoculum containing ~ 6 log_10_ CFU/mL culture. The mating experiments were conducted in 8-mL volumes of LB broth by mixing the donor and recipient cell suspensions in a 1:1 ratio and incubating for 24 h at 37 °C with or without the SIC of phytochemicals. The donor and recipient bacteria were also grown individually for 24 h with or without the phytochemicals. Transconjugants were recovered after serial dilution and plating on TSA containing 100 μg/mL of ampicillin and 50 μg/mL of nalidixic acid by incubating at 37 °C for 24 h. The donor cells were recovered on TSA-ampicillin (100 μg/mL) plates, and the recipients were enumerated on TSA-nalidixic acid (50 μg/mL).

Horizontal gene transfer (HGT) frequency was calculated as the ratio of the number of transconjugant CFU per mL to the number of recipient CFU per mL, as described previously (47–49):


$$\mathrm{HGT}\;\text{frequency}=\frac{{\mathrm{CFU}}_{T}}{{\mathrm{CFU}}_{R}}$$

where CFU_T_ represents the concentration of transconjugants and CFU_R_ represents the concentration of recipient cells, respectively, expressed as CFU/mL.

Log₁₀ HGT frequency was calculated according to:


$$\log_{10}(\mathrm{HGT})=\log_{10}({\mathrm{CFU}}_{T})-\log_{10}({\mathrm{CFU}}_{R})$$

Where CFU_t_ and CFUᵣ are expressed as absolute CFU/mL values. The log₁₀-transformed values of CFU were used only for data presentation and calculation of log₁₀ HGT frequency.

### 2.5 Manure sample preparation for conjugation experiments

Fresh chicken manure was collected from the University of Connecticut Poultry Farm. Since *S*. Heidelberg (donor) and *E. coli* nalidixic acid resistant (recipient) were separately incubated in 30 mL volumes of LB broth at 37 °C for 18 h to form stationary-phase cultures containing approximately 8 log_10_ CFU/mL. A 1 mL aliquot from each stationary-phase culture was serially diluted twice in 9 mL volumes of LB broth to form an inoculum containing approximately 6 log_10_ CFU/mL culture. The manure samples were collected immediately after being deposited onto the bedding and transferred to the laboratory in whirl-pack bags. The samples were sterilized by autoclaving at 121 °C for 15 min to eliminate background fecal microbiota. Manure samples (2.5 g) were then mixed with 8 mL of Maximum recovery diluent (MRD, Oxoid, Cheshire). Donor and recipient cell suspensions in 1:1 ratios were added to 8 mL of MRD and incubated for 24 h at 25 °C with or without the SIC of phytochemicals. The donor and recipient strains were also grown independently for 24 h with or without the aforementioned phytochemicals. Transconjugants were then recovered after serial dilution and plating on TSA containing 100 μg/mL of ampicillin and 50 μg/mL of nalidixic acid by incubating at 37 °C for 24 h. The donor cells were recovered on TSA-ampicillin (100 μg/mL) plates, and the recipients were enumerated on TSA-nalidixic acid (50 μg/mL).

### 2.6 Conjugation experiments in water samples

Spring water samples were collected in sterile tubes from a nearby natural spring. The water samples were streaked on TSA and XLD plates to determine the initial bacterial count and *Salmonella,* if any. Overnight cultures of *Salmonella* and *E. coli* OP50 were serially diluted to form an inoculum containing approximately 7 log_10_ CFU/mL. Conjugation experiments in water were conducted in 9.8 mL of spring water by mixing 100 μL each of the donor and recipient cell suspensions (1:1 ratio) and incubated for 24 h at 25 °C with or without the SIC of phytochemicals. Conjugation frequencies were determined as described previously.

### 2.7 *Ex vivo* conjugation study in chickens

The efficacy of TC and CR in reducing HGT of *β*-lactamase gene (*bla*_TEM_) from *S*. Heidelberg to *E. coli* in an *ex vivo* chicken model was determined, as previously described (85). Animal use was approved by the University of Connecticut Institutional Animal Care and Use Committee (IACUC protocol # A18-036).

A total of two hundred fifty-two, day-old, *Salmonella*-free, Cornish cross broiler birds were obtained from Myers Poultry Farm (South Fork, PA), housed at the University of Connecticut’s Spring Hill Poultry Isolation facility, and provided ad libitum feed and water. All birds were fed a standard commercial starter–grower feed (Home Fresh Multi-Flock Chick N Game Starter/Grower, Blue Seal, Litchfield, IL) that was completely free of antibiotics or medications. The precise guaranteed analysis and nutrient composition of this commercial basal diet are listed in Supplementary Table S1. Upon arrival (Day 1), the birds were randomly allocated into seven initial treatment groups (*n* = 36 birds per group) as part of a larger multi-endpoint study. The birds were randomly allocated to their respective treatment groups, with the birds of each group housed together in a single, separate floor pen to prevent cross-contamination between treatments. The birds were provided with water and experimental diets (treatment and control) ad libitum for a period of 16 days. For the scope of the present manuscript, data from five distinct dietary treatment groups are reported: a control diet, two diets supplemented with TC (0.5 and 1.0% v/w), one diet supplemented with CR (0.125% v/w), and a diet supplemented with a combination of TC and CR (0.25% v/w each) (Table 1). These two phytochemicals were chosen due to their ability to reduce HGT *in vitro* and their proven palatability and efficacy in chickens at these specific concentrations, as established by previous literature (39, 50, 83, 85). To ensure uniform distribution and prevent compound volatilization, the specified volumes of pure liquid phytochemicals were measured daily using a syringe, added directly to the solid commercial basal feed, and thoroughly mixed by hand prior to provisioning. All in-feed dosages are expressed as volume-to-weight percentage (% v/w, mL of pure compound per 100 g of feed) rather than body-weight parameters, representing standard dietary inclusion rates for poultry feed additive evaluations. Birds were kept at an initial temperature of 32.2 °C for the first week, and the temperature was reduced to 26.6 °C the following week until day 16, along with a 20-h light/4-h dark cycle.

**Table 1:** Treatment groups in the *ex vivo* study in chickens.

Bird groups	Treatment
Negative control	Birds fed with control diet, non-inoculated
Positive control	Birds fed with control diet, Inoculated with SH (donor) and *E. coli* (recipient)
Treatment 1 (T1)	Birds fed 0.5%TC, Inoculated with SH (donor) and *E. coli* (recipient)
Treatment 2 (T2)	Birds fed 1%TC, Inoculated with SH (donor) and *E. coli* (recipient)
Treatment 3 (T3)	Birds fed 0.125% CR, Inoculated with SH (donor) and *E. coli* (recipient)
Treatment 4 (T4)	Birds fed 0.25%TC and 0.25% CR, Inoculated with SH (donor) and *E. coli* (recipient)

All the birds were fed with the experimental diet starting from day 1 until day 16 (end timepoint). SH, *S*. Heidelberg.

On day 16, a subset of eight birds from each reported group (*n* = 8) was randomly selected and euthanized by carbon dioxide asphyxiation for separate collection of paired ceca to perform the *ex vivo* conjugation assay. The patent end of each cecum was carefully knotted using sterile suture material and placed in a petri dish containing 10 mL of phosphate-buffered saline (pH 7.2) until further processing. Lawn cultures of donor *S*. Heidelberg and recipient *E. coli* OP 50 were grown on XLD (with ampicillin and streptomycin) and Mac Conkey agar (with nalidixic acid and streptomycin), respectively, for 24 h at 41 °C (normal chicken body temperature), and later harvested in 20 mL of PBS to obtain 8 log_10_ CFU/mL of donor and recipient bacteria. Each 100 μL donor *S*. Heidelberg and recipient *E. coli* culture suspension (in PBS) was separately inoculated into the individual cecal loop using a tuberculin syringe. The cecal samples were incubated for four hours at 41 °C. Following incubation, cecal samples were minced, homogenized in 25 mL of PBS, and plated on (a) selective XLD agar containing ampicillin (100 μg/mL)—streptomycin (10 μg/mL) to obtain donor *Salmonella* population, (b) Mac Conkey agar containing nalidixic acid (50 μg/mL)—streptomycin (10 μg/mL) to obtain the recipient *E. coli* population, and (c) Mac Conkey agar containing nalidixic acid (50 μg/mL) –streptomycin (10 μg/mL)–ampicillin (100 μg/mL) to obtain the transconjugant population. As differences in the microbiome influence the colonization of donor and recipient bacteria, a bias arises when evaluating HGT frequency based on recipient bacteria alone. Thus, the impact of both the donor and recipient bacterial colonization was considered. To correct for variations in the colonization by donor and recipient bacterial populations in the chicken study, the HGT frequency was calculated as the ratio of the number of transconjugant CFU per g to the number of recipient CFU per g.

### 2.8 Colony polymerase chain reaction

The transconjugant bacterial DNA was examined for the presence of *bla*_TEM_ encoding the *β*-lactamase gene using a colony polymerase chain reaction. The recipient *E. coli* was confirmed by amplifying the gene coding for universal stress protein A (*usp*A), which is specific for *E. coli* (51), whereas donor *Salmonella* was differentiated by the presence of *bla*_TEM_ and *inv*A genes, which encode beta-lactamase and *Salmonella*-specific invasion protein, respectively (52, 53). The transconjugants were selected on Mac Conkey agar containing ampicillin (100 μg/mL) and nalidixic acid (50 μg/mL). The donor and recipient bacteria were grown separately on XLD agar containing ampicillin (100 μg/mL) and Mac Conkey agar with nalidixic acid (50 μg/mL), respectively, for 24 h at 37 °C, and one colony from each plate was picked and resuspended in 500 μL of sterile distilled water. The cells were lysed by heating at 95 °C for 10 min, and cellular debris was removed by centrifugation at 16,000 × g for 2 min (54). The *bla*_TEM_ in the supernatant was amplified by PCR, as described by Walsh et al. (46) using a Bio-Rad C1000 touch thermocycler. The nucleotide sequences of the primers and the cycling conditions of the primers are shown in Table 2. The resulting products were run on a 1% agarose gel stained with 0.2 μg/mL ethidium bromide and visualized using a gel documentation system (Bio-Rad, Hercules, CA).

**Table 2:** Thermocycler amplification conditions for the genes used for colony PCR.

Gene	Denaturation	Annealing	Extensions	No. of cycles	Primers
*bla*_TEM_	94 °C, 2 min	54 °C, 1 min	72 °C, 30 s	30	Fwd5’TTG GGT GCA CGA GTG GGT TA-3’ Rev5’TAA TTG TTG CCG GGA AGC TA3’
*inv*A	94 °C, 1 min	53 °C, 2 min	72 °C, 3 min	35	Fwd5’GTGAAATTATCGCCACGTTCGGGCAA3’ Rev5’TCATCGCACCGTCAAAGGAACC3’
*usp*A	94 °C, 2 min	70 °C, 1 min	72 °C, 1 min	30	Fwd5’CCGATACGCTGCCAATCAGT3’ Rev5’ACGCAGACCGTAGGCCAGAT3’

### 2.9 Whole genome sequencing and confirmation for the presence of plasmids and antimicrobial resistance genes

To determine if the recipient *E. coli* acquired the plasmid containing the *bla*_TEM_ gene from the donor Salmonella during conjugation studies, WGS was performed on the isolates. DNA extraction was conducted using representative colonies from the original donor *S.* Heidelberg, the clean recipient *E. coli*, and the resulting transconjugant *E. coli*. Four transconjugant colonies were randomly selected from selective agar plates (following LB broth conjugation experiments), individually resuspended in 200 μL sterile PBS, and subjected to qPCR analysis targeting the *bla*_TEM_ gene to confirm pure cultures and verify plasmid acquisition prior to sequencing. DNA extraction was conducted using pooled DNA from these 4 qPCR-verified transconjugant isolates, the original donor *S*. Heidelberg, and the recipient *E. coli*. Total genomic and plasmid DNA of the donor bacteria, recipient *E. coli*, and transconjugant *E. coli* were extracted from overnight cultures grown on TSA plates with respective antibiotics using Qiagen’s QIAamp BiOstic Bacteremia DNA kit and QIAprep Spin Miniprep Kit (Qiagen, Germantown, MD), respectively. The extracted DNA was quantified using the nanodrop (Thermo Scientific, Waltham, MA), as per the manufacturer’s instructions. The whole genome sequencing was performed by next-gen sequencing using Illumina NovaSeq platform with NovaSeq 6000SP reagent. Paired-end sequence reads were quality checked, assembled and annotated using FastQC, Unicycler and Prokka, respectively using the Galaxy platform. Furthermore, AR genes and plasmid types were detected using the CGE Resfinder database (with a similarity cut-off of 97%) and PlasmidFinder database (with a similarity cut-off of 100%) respectively.

### 2.10 Effect of phytochemicals on transfer genes involved in bacterial conjugation

The effect of phytochemicals on the transcription of *S*. Heidelberg transfer genes involved in conjugation was studied using real-time quantitative PCR (RT-qPCR). The specific primers for the conjugation transfer genes were designed using the primer Basic Logic Alignment Search Tool (BLAST) (Table 3) using IncI plasmid as a template. The 16S rRNA gene in *Salmonella* was used as the endogenous control. *S.* Heidelberg was grown in the presence of SIC of each phytochemical in LB broth and cells were collected after 6 h of incubation at 37 °C. The RNA was extracted using a Pure Link RNA mini kit (Thermo Fischer Scientific, Waltham, MA), subjected to DNase I digestion using Turbo DNase I (Ambion, Waltham, MA) and quantified using a nanodrop. The RNA content was normalized in all treatments, and cDNA was synthesized using the Bio-Rad iScript cDNA synthesis kit (Bio-Rad, Hercules, CA) using the Bio-Rad C1000 touch thermocycler. RT-qPCR analysis of the genes critical for conjugation was performed using published primers normalized against 16S rRNA gene expression. Twenty-microliter reactions were performed in triplicate using iTaq SYBR (Bio-Rad, Hercules, CA). The relative fold change in gene expression was calculated using the 2^−ΔΔCt^ method (55).

**Table 3:** Complete list of primers used for RT-qPCR.

Primer	5′ to 3’ sequence
*16 s* F	CGTGTTGTGAAATGTTGGGTTAA
*16 s* R	CCGCTGGCAACAAAGGATAA
*fin*O F ST	TGACCATGGACGAATGGCTC
*fin*O R ST	TGACCATCCAGGCCATAACG
*fin*O F SH	ACCATGGACGAATGGCTCTG
*fin*O R SH	AATGTCACCAGTCTGCGACC
*tra*G F	CTGTCCATAACGACGGGTTC
*tra*G R	TCGGATAAAAGCGGAATCAC
*tra*H F	GGACGTGAAGGTTGACTGGT
*tra*H R	GACTGGGAAGGTGATGCAAT
*tra*I F	TTGTCTTCCTTCCTGCCATC
*tra*I R	TGAACGCTTTGTCAGCAATC
*tra*J F	GCTTTACGACCACCGTCATT
*tra*J R	CCTGTCATCAGGGATTCGAT
*tra*M F	AATATTCGCGCTCCACATTC
*tra*M R	AACAGCGGGCAAATAATGTC
*tra*N F	GGTGACAGTGGGCTGAAGAA
*tra*N R	ACGGTTCACCACATCCCTTC
*tra*R F	TCGACATTGCGAACCATATC
*tra*R R	GCCGGAGCAAACTGACTAAG

### 2.11 Effect of phytochemicals in plasmid curing

The experiment to determine the plasmid curing (plasmid removal) from *Salmonella* donor strain was performed as described previously (56). In brief, donor *Salmonella* was grown overnight at 37 °C in 10 mL of LB broth with and without the SIC of phytochemicals. Since ethidium bromide was shown to have plasmid curing properties, 100 μg/mL of ethidium bromide was used as a positive control (56). A volume of 0.5 mL of 10-fold serial dilutions of overnight cultures was grown on TSA agar without antibiotics and incubated overnight at 37 °C. The isolated colonies were replicate-plated on TSA agar and TSA agar containing 100 μg/mL of ampicillin using Replica Plating Tool (Scienceware, Bel-Art Products) and incubated for 24 h at 37 °C before colony counting. The colonies that failed to grow on TSA agar containing antibiotics were considered putative plasmid-cured colonies.

### 2.12 Electron microscopy

To visualize the effect of phytochemicals on *Salmonella* pilus formation, transmission electron microscopic (TEM) analysis of treated cells was performed. Donor *S*. Heidelberg was grown overnight at 37 °C in LB broth with and without the phytochemicals. The cells were pelleted after centrifugation at 3000 g for 15 min. The pellet was resuspended in a fixative solution containing 1.5% glutaraldehyde, 3 mM magnesium chloride, and 0.12 M phosphate buffer for 1 h. Briefly, 3 μL of the sample was absorbed into a carbon-coated copper grid and washed with 100 μL of 0.5% of uranyl acetate. TEM images were taken using an FEI Tecnai TM microscope (FEI, Hillsboro, OR).

### 2.13 Statistical analysis

For the *in vitro* LB broth, water and manure matrix trials, a completely randomized design was followed, and an individual tube was considered the experimental unit. For the *ex vivo* chicken study, each individual bird served as the independent experimental unit (*n* = 8) per group. The two distinct ceca harvested from each bird were inoculated and processed separately, with their values averaged to represent the final data point for that specific experimental unit. Two samples were used for each treatment and control in the matrix trials, and the experiments were repeated six times. Duplicate plates were used as technical replicates for each dilution to reduce personnel error while plating. Transconjugant, donor *Salmonella* and recipient *E. coli* counts were used to calculate the HGT frequencies separately for each experimental unit. All the data reported are presented as mean ± standard error of means (SEM).

Statistical analysis was conducted using the non-parametric Kruskal-Wallis test, with post-hoc analysis corrected for multiple comparisons using Dunn’s multiple comparison test. A significance level was set at *p* ≤ 0.05. All statistical analyses were conducted, and the graphs were plotted utilizing Prism 8 (GraphPad Software Inc., San Diego, CA). These non-parametric methods were selected because the microbial count and conjugation frequency data exhibited non-normal distributions and unequal variances across treatment groups, violating the assumptions required for standard parametric analysis. A significance level was set at *p* ≤ 0.05.

## 3 Results

### 3.1 PCR confirmation of conjugal transfer of *bla*_TEM_

Two different approaches were utilized to demonstrate the transfer of the *bla*_TEM_ gene from donor *Salmonella* to recipient *E. coli*. Firstly, selective plating of transconjugants on Mac Conkey agar containing ampicillin and nalidixic acid was used for phenotypic confirmation of *E. coli* that acquired ampicillin resistance from the donor *Salmonella*. Secondly, genotypic analysis of transconjugant colonies was performed to confirm the presence of the *bla*_TEM_ gene in transconjugant *E. coli*. The donor *Salmonella* was confirmed by the presence of *inv*A (284 bp) and *bla*_TEM_ (500 bp) genes, whereas the recipient *E. coli* was confirmed by the presence of *uspA* (884 bp). As expected, the transconjugant colonies showed both the *bla*_TEM_ gene and the *usp*A gene (Figure 1). Collectively, these results verified the occurrence of conjugal transfer of the *bla*_TEM_ gene from *Salmonella* to *E. coli.*

**Figure 1:**
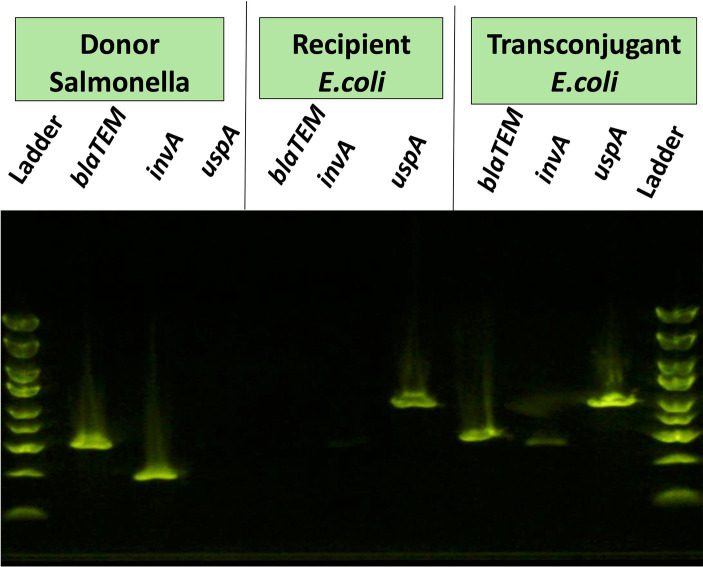
Colony PCR products from donor *Salmonella*, recipient *E. coli*, and transconjugant *E. coli*. Lane 1, 100 bp DNA ladder; Lanes 2 to 4, PCR amplicons from donor *Salmonella* (*bla*_TEM_, *invA*, *uspA*); Lanes 5 to 7, PCR amplicons from recipient *E. coli* (*bla*_TEM_, *invA*, *uspA*); Lanes 8 to 10, PCR amplicons from transconjugant *E. coli* (*bla*_TEM_, *invA*, *uspA*); Lane 11, 100 bp DNA ladder. The full-length, uncropped gel image corresponding to this figure is provided in Supplementary Figure S1. No lane rearrangement or selective editing was performed.

### 3.2 Whole-genome sequencing of donor, recipient, and transconjugant *Escherichia coli*

WGS analyses using Resfinder and PlasmidFinder detected the presence of AR genes and plasmid, respectively in the donor, and transconjugant *E. coli*, as listed in Table 4. The donor *S.* Heidelberg and the transconjugant *E. coli* carried IncI1-I(Alpha) plasmid containing the beta-lactamase gene (*bla*_TEM-1B_). However, no plasmids and *bla*_TEM-1B_ were identified in recipient *E. coli*, which further confirms that the transconjugant *E. coli* acquired the *bla*_TEM-1B_ gene from the donor *Salmonella* via horizontal gene transfer (Table 4).

**Table 4:** AMR genes and plasmids detected in Resfinder and PlasmidFinder, respectively.

Organism	Plasmid present	AR phenotype	AR genes
Genomic EC	-	Nalidixic acid	*gyr*A
		Doxycycline, tetracycline, minocycline	*tet*(B)
Genomic SH	IncI1-I(Alpha)	Apramycin, gentamicin, tobramycin, dibekacin, netilmicin, sisomicin	*aac*(3)-IId
		Amikacin, tobramycin	*aac*(6′)-Iaa
		Amoxicillin, ampicillin, cephalothin, piperacillin, ticarcillin	*bla*_TEM-1B_
		Fosfomycin	*fos*A7
Genomic SHEC	IncI1-I(Alpha)	Apramycin, gentamicin, tobramycin, dibekacin, netilmicin, sisomicin	*aac*(3)-IId
		Nalidixic acid, ciprofloxacin	*gyr*A
		Amoxicillin, ampicillin, cephalothin, piperacillin, ticarcillin	*bla*_TEM-1B_
		Doxycline, tetracycline, minocycline	*tet*(B)
Plasmid EC	-	-	*-*
Plasmid SH	IncI1-I(Alpha)	Amikacin, tobramycin	*aac*(6′)-Iaa
		Apramycin, gentamicin, tobramycin, dibekacin, netilmicin, sisomicin	*aac*(3)-IId
		Amoxicillin, ampicillin, cephalothin, piperacillin, ticarcillin	*bla*_TEM-1B_
		Fosfomycin	*fos*A7
Plasmid SHEC	IncI1-I(Alpha)	Apramycin, gentamicin, tobramycin, dibekacin, netilmicin, sisomicin	*aac*(3)-IId
		Nalidixic acid	*gyr*A
		Amoxicillin, ampicillin, cephalothin, piperacillin, ticarcillin	*bla*_TEM-1B_
		Tetracycline, doxycycline, minocycline	*tet*(B)

Both plasmid and genomic DNA were separately isolated and the whole genome sequenced. EC, *E. coli*; SH, *S*. Heidelberg; SHEC, transconjugant *E. coli* when donor *S.* Heidelberg was used for conjugation.

### 3.3 Phytochemicals reduced transconjugant population in LB broth

The SIC of phytochemicals against *Salmonella* and *E. coli* were separately determined in LB broth, water and manure and are presented in Table 5. In LB broth, the untreated control supported high levels of plasmid transfer, generating 6.03 log₁₀ CFU/mL of transconjugant *E. coli* after 24 h (Table 6). Treatment with the phytochemicals resulted in varying degrees of inhibition of conjugation.

**Table 5:** Subinhibitory concentrations (SIC) of TC, CR, CA and BR against *S*. Heidelberg and *E. coli* in LB broth, chicken manure, and water.

Phytochemicals	LB broth	Chicken manure	Water
Trans-cinnamaldehyde (TC)	0.15 mM (0.020%)	0.15 mM (0.02%)	0.0113 mM (0.015%)
Carvacrol (CR)	0.07 mM (0.01%)	0.332 mM (0.05%)	0.008 mM (0.0125%)
Caprylic acid (CA)	0.49 mM (0.07%)	1.04 mM (0.15%)	0.0069 mM (0.01%)
β-resorcylic acid (BR)	0.32 mM (0.05%)	0.973 mM (0.15%)	0.0048 mM (0.0075%)

**Table 6:** Effect of TC, CR, CA and BR on bacterial counts after 24 h incubation with the donor *S.* Heidelberg and *E. coli* in LB broth (log_10_ CFU/mL).

Treatment	Recipient *E. coli* (log₁₀ CFU/mL)	Donor *S*. Heidelberg (log₁₀ CFU/mL)	Transconjugants (log₁₀ CFU/mL)	HGT frequency (ratio)	log₁₀ HGT frequency
Control	8.49 ± 0.029	8.72 ± 0.025	6.03 ± 0.037	3.47 × 10^−3^	−2.46
TC	8.43 ± 0.033	8.65 ± 0.053	5.26 ± 0.012	6.76 × 10^−4^	−3.17^*^
CR	7.95 ± 0.014	8.23 ± 0.045	1.00 ± 0.000	1.12 × 10^−7^	−6.95^*^
CA	8.17 ± 0.099	8.18 ± 0.037	4.52 ± 0.216	2.24 × 10^−4^	−3.65^*^
BR	7.90 ± 0.102	8.61 ± 0.067	4.09 ± 0.010	1.55 × 10^−4^	−3.81^*^
DMSO	8.20 ± 0.051	8.91 ± 0.001	6.01 ± 0.011	6.46 × 10^−3^	−2.19

Data are mean ± SEM. * indicates a significant difference from the untreated control (Kruskal–Wallis with Dunn’s multiple comparison test, *p* ≤ 0.05). TC, trans-cinnamaldehyde; CR, Carvacrol; CA, Caprylic acid; BR, β-resorcylic acid; DMSO, dimethyl sulfoxide.

Among all compounds, carvacrol (CR) exerted the strongest inhibitory effect, reducing transconjugants to 1.00 log₁₀ CFU/mL, corresponding to an HGT frequency of 1.12 × 10^−7^ (log₁₀ = −6.95) (*p <* 0.05), the lowest observed across treatments. *β*-Resorcylic acid (BR) also significantly suppressed plasmid transfer, lowering transconjugant levels to 4.09 log₁₀ CFU/mL with an HGT frequency of 1.55 × 10^−4^ (log₁₀ = −3.81) (*p <* 0.05). Trans-cinnamaldehyde (TC) and caprylic acid (CA) showed more moderate reductions. TC decreased transconjugants to 5.26 log₁₀ CFU/mL (HGT = 6.76 × 10^−4^), while CA yielded 4.52 log₁₀ CFU/mL (HGT = 2.24 × 10^−4^) (*p <* 0.05). These reductions were less pronounced than those observed for CR and BR but still indicated measurable decreases in conjugation efficiency. The DMSO solvent control did not inhibit plasmid transfer; transconjugant counts (6.01 log₁₀ CFU/mL) and HGT frequency (6.46 × 10^−3^) were comparable to or slightly higher than the untreated control (*p* > 0.05). Collectively, these results demonstrate that CR was the most potent inhibitor of HGT in LB broth, followed by BR, while TC and CA exhibited moderate inhibitory effects, and DMSO yielded no effect.

### 3.4 Phytochemicals reduced the transconjugant population in chicken manure

In untreated chicken manure, plasmid-mediated ampicillin resistance transferred efficiently from *Salmonella* to *E. coli*, generating 6.99 log₁₀ CFU/mL of transconjugants after 24 h at 25 °C, while recipient populations remained high at 8.73 log₁₀ CFU/mL (Table 7). Treatment with phytochemicals significantly reduced plasmid transfer. Carvacrol (CR) produced the strongest inhibitory effect, lowering transconjugant levels to 3.73 log₁₀ CFU/mL and reducing HGT frequency from 1.82 × 10^−2^ in the control to 5.89 × 10^−5^ (log₁₀ = −4.23) (*p <* 0.05). *β*-Resorcylic acid (BR) also markedly suppressed plasmid transfer, reducing transconjugants to 4.33 log₁₀ CFU/mL and decreasing HGT frequency to 2.40 × 10^−4^ (log₁₀ = −3.62) (*p <* 0.05).

**Table 7:** Effect of TC, CR, CA and BR on bacterial counts after 24 h incubation in chicken manure with the donor *S.* Heidelberg and recipient *E. coli* (log_10_ CFU/mL).

Treatment	Recipient *E. coli* (log₁₀ CFU/mL)	Donor *S*. Heidelberg (log₁₀ CFU/mL)	Transconjugants (log₁₀ CFU/mL)	HGT frequency (ratio)	log₁₀ HGT frequency
Control	8.73 ± 0.060	8.80 ± 0.035	6.99 ± 0.051	1.82 × 10^−2^	−1.74
TC	8.42 ± 0.029	8.55 ± 0.024	5.87 ± 0.078	2.82 × 10^−3^	−2.55^*^
CR	7.96 ± 0.157	8.17 ± 0.054	3.73 ± 0.166	5.89 × 10^−5^	−4.23^*^
CA	8.33 ± 0.031	8.40 ± 0.043	5.69 ± 0.096	2.29 × 10^−3^	−2.64^*^
BR	7.95 ± 0.151	8.00 ± 0.085	4.33 ± 0.253	2.40 × 10^−4^	−3.62^*^
DMSO	8.91 ± 0.056	8.37 ± 0.062	6.54 ± 0.087	4.27 × 10^−3^	−2.37

Data are mean ± SEM. * indicates a significant difference from the untreated control (Kruskal–Wallis with Dunn’s multiple comparison test, *p* ≤ 0.05). TC, trans-cinnamaldehyde; CR, Carvacrol; CA, Caprylic acid; BR, β-resorcylic acid; DMSO, dimethyl sulfoxide.

Moderate reductions were observed with trans-cinnamaldehyde (TC) and caprylic acid (CA), which yielded 5.87 log₁₀ and 5.69 log₁₀ CFU/mL of transconjugants, respectively. Their corresponding HGT frequencies (2.82 × 10^−3^ and 2.29 × 10^−3^) were lower than the control but did not match the magnitude of inhibition observed with CR and BR. Similar to LB broth, the DMSO solvent control showed no inhibitory effect, producing 6.54 log₁₀ CFU/mL of transconjugants with an HGT frequency of 4.27 × 10^−3^, comparable to or slightly above the untreated control. These findings demonstrate that CR was the most effective inhibitor of HGT in poultry manure, followed by BR, whereas TC and CA showed weaker inhibition, and DMSO did not affect plasmid transfer.

### 3.5 Phytochemicals reduced transconjugant population in spring water

The effect of the phytochemicals on plasmid-mediated transfer of ampicillin resistance in spring water is summarized in Table 8. In the untreated control, 4.17 log₁₀ CFU/mL of transconjugant *E. coli* were recovered after 24 h at 25 °C, from a recipient population of 7.78 log₁₀ CFU/mL, indicating efficient conjugation under these conditions.

**Table 8:** Effect of TC, CR, CA and BR on bacterial counts after 24 h incubation in water with the donor *S.* Heidelberg and recipient *E. coli* (log_10_ CFU/mL).

Treatment	Recipient *E. coli* (log₁₀ CFU/mL)	Donor *S.* heidelberg (log₁₀ CFU/mL)	Transconjugants (log₁₀ CFU/mL)	HGT frequency (ratio)	log₁₀ HGT frequency
Control	7.78 ± 0.152	8.05 ± 0.166	4.17 ± 0.168	2.45 × 10^−4^	−3.61
TC	8.01 ± 0.029	8.17 ± 0.039	3.70 ± 0.198	4.90 × 10^−5^	−4.31^*^
CR	7.75 ± 0.089	8.03 ± 0.058	3.32 ± 0.188	3.72 × 10^−5^	−4.43^*^
CA	7.82 ± 0.066	8.02 ± 0.063	3.23 ± 0.198	2.57 × 10^−5^	−4.59^*^
BR	7.88 ± 0.054	8.01 ± 0.166	3.59 ± 0.174	5.13 × 10^−5^	−4.29^*^
DMSO	7.74 ± 0.039	8.07 ± 0.110	4.24 ± 0.002	3.55 × 10^−4^	−3.50

Data are mean ± SEM. * indicates a significant difference from the untreated control (Kruskal–Wallis with Dunn’s multiple comparison test, *p* ≤ 0.05). TC, trans-cinnamaldehyde; CR, carvacrol; CA, caprylic acid; BR, β-resorcylic acid; DMSO, dimethyl sulfoxide.

All phytochemicals reduced transconjugant populations to varying degrees, with carvacrol (CR) and caprylic acid (CA) producing the greatest reductions. CR lowered transconjugants to 3.32 log₁₀ CFU/mL, while CA reduced them further to 3.23 log₁₀ CFU/mL. Both treatments significantly decreased HGT frequency to 3.72 × 10^−5^ (CR) and 2.57 × 10^−5^ (CA), corresponding to log₁₀ values of −4.43 and −4.59, respectively (*p <* 0.05).

Moderate reductions were observed with trans-cinnamaldehyde (TC) and β-resorcylic acid (BR), which produced transconjugant counts of 3.70 and 3.59 log₁₀ CFU/mL, respectively. The HGT frequencies (4.90 × 10^−5^ for TC and 5.13 × 10^−5^ for BR) remained lower than the control but were less pronounced than those achieved with CR and CA.

The DMSO solvent control exhibited no inhibitory effect, yielding 4.24 log₁₀ CFU/mL of transconjugants and an HGT frequency of 3.55 × 10^−4^, closely matching the untreated control. Overall, these results indicate that CR and CA were the most effective inhibitors of plasmid transfer in spring water, followed by TC and BR, whereas DMSO had no impact on conjugation efficiency.

### 3.6 Efficacy of phytochemicals *ex vivo*

The effect of in-feed phytochemicals on the dissemination of ampicillin resistance was evaluated *ex vivo* using cecal contents collected on day 16. Following inoculation with 8 log₁₀ CFU/mL each donor *S.* Heidelberg and recipient *E. coli*, the recovered bacterial populations in the control group averaged 5.72 log₁₀ CFU/g (donor) and 6.57 log₁₀ CFU/g (recipient) after 4 h of incubation (Table 9). Transconjugant levels in the positive control reached 7.01 log₁₀ CFU/g, resulting in a high HGT frequency of 2.75 × 10^0^ (log₁₀ = +0.44).

**Table 9:** Effect of in-feed phytochemical supplementation on bacterial counts in cecal content after 4 h incubation with the donor *S.* Heidelberg, and recipient *E. coli* (log_10_ CFU/g) on day 16 *ex vivo*.

Treatment	Recipient (*E. coli*) (log₁₀ CFU/g)	Donor (*S.* Heidelberg) (log₁₀ CFU/g)	Transconjugants (log₁₀ CFU/g)	HGT frequency (ratio)	log₁₀ HGT frequency
Control	6.57 ± 0.214	5.72 ± 0.139	7.01 ± 0.115	2.75 × 10^0^	+0.44
T1	6.88 ± 0.089	5.61 ± 0.099	6.53 ± 0.185	4.47 × 10^−1^	−0.35^*^
T2	7.00 ± 0.082	5.00 ± 0.194	6.65 ± 0.189	4.47 × 10^−1^	−0.35^*^
T3	7.01 ± 0.112	5.41 ± 0.098	6.55 ± 0.167	3.47 × 10^−1^	−0.46^*^
T4	7.10 ± 0.074	5.64 ± 0.182	7.00 ± 0.086	7.94 × 10^−1^	−0.10^*^

On day 16, paired ceca were collected from birds and inoculated with equal populations of donor *S.* Heidelberg and recipient *E. coli* culture suspension (in PBS) into the individual cecal loop to perform the ex vivo conjugation assay. Data are mean ± SEM. * indicates a significant difference from the untreated control (Kruskal–Wallis with Dunn’s multiple comparison test, *p* ≤ 0.05).

Across all phytochemical-supplemented groups (T1–T4), transconjugant counts remained within a similar range (6.53–7.00 log₁₀ CFU/g). However, notable reductions in HGT frequency were observed in several treatment groups. The strongest reduction occurred in birds fed 0.5% TC (T1) and 1% TC (T2), both yielding an HGT frequency of 4.47 × 10^−1^ (log₁₀ = −0.35), compared with 2.75 × 10^0^ in the control. Birds receiving 0.125% CR (T3) also showed decreased HGT frequency (3.47 × 10^−1^, log₁₀ = −0.46). The TC–CR combination group (T4) exhibited a more modest reduction in HGT frequency (7.94 × 10^−1^, log₁₀ = −0.10), remaining higher than other treatments but still below the control. Overall, while transconjugant counts did not differ significantly among groups, TC-based treatments (0.5 and 1% TC) and CR supplementation significantly lowered HGT frequency, indicating that these phytochemicals reduced conjugation efficiency without reducing total transconjugant population size.

### 3.7 Phytochemicals modulated conjugation genes

To delineate the effect of phytochemicals on the conjugative mechanism, real-time quantitative PCR (RT-qPCR) analysis of genes critical for the regulation of donor bacterial conjugation was performed. The RT-qPCR analysis revealed that the phytochemical treatments altered the expression of core genes associated with conjugation in donor *Salmonella* as compared to the control. It was found that all the tested phytochemicals significantly downregulated (1-2-fold) a majority of the *tra* genes (*tra*G, *tra*H, *tra*I, *tra*J, *tra*M, *tra*N and *tra*R, Figure 2, *p <* 0.05). Further, BR significantly upregulated the transcription of *fin*O gene when compared to the control (*p <* 0.05). These findings indicate that the phytochemicals adversely affected the conjugative machinery of *Salmonella*.

**Figure 2:**
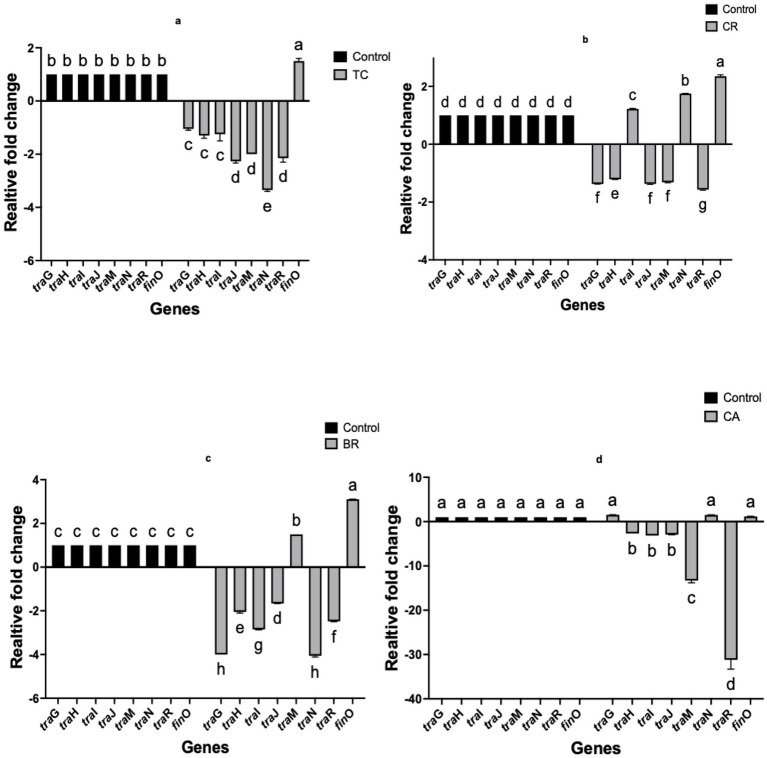
Effect of trans-cinnamaldehyde (TC) **(a)**, Carvacrol (CR) **(b)**, β-resorcylic acid (BR) **(c)**, Caprylic acid (CA) **(d)** on *Salmonella* conjugation-associated genes. LB broth with or without SIC concentrations of the phytochemicals was inoculated with *S.* Heidelberg and incubated for 6 h. *, treatments differed significantly from the control group (*p* < 0.05).

### 3.8 Electron microscopy

The surface pilus phenotype of phytochemical-treated *Salmonella* was assessed using negative-stain EM for observation of any reduction in the pilus production on the cell surface. As evident in Figures 3a–e, there was a decreased presence of surface pili on *Salmonella* cells treated with the phytochemicals in comparison to the control. These results corroborate the RT-qPCR results that demonstrated reduced expression of genes involved in the pilus biogenesis pathway.

**Figure 3:**
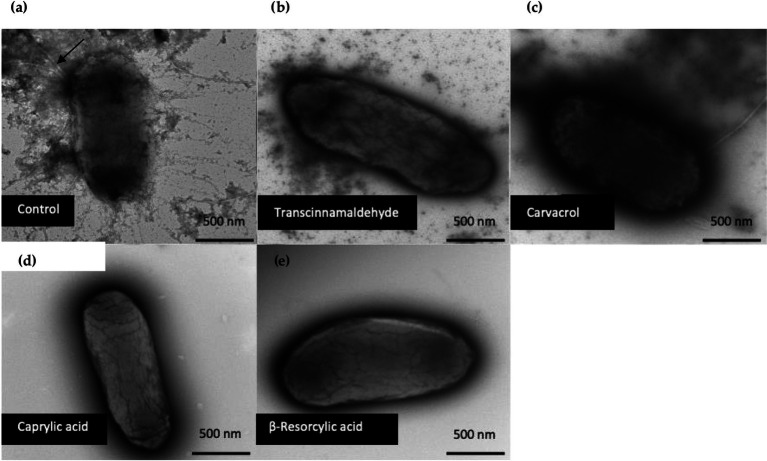
Negative staining TEM image of *S*. Heidelberg **(a–e)**. Phytochemical-treated *Salmonella* showing the reduced presence of surface pilus when compared to the control untreated *Salmonella* **(b–e)**. Control **(a)**, and *S*. Heidelberg treated with TC **(b)**, CR **(c)**, CA **(d)**, and BR **(e)**. The black arrow **(a)** shows the presence of pilus.

### 3.9 Plasmid curing

No plasmid curing activity was observed when donor *Salmonella* was exposed to any of the phytochemicals. This suggests that the plasmid containing *bla*_TEM-1B_ is stable in *S.* Heidelberg. The mechanism of inhibition of conjugation brought by the phytochemicals was not due to the loss of the plasmid bearing *bla*_TEM-1B_.

## 4 Discussion

In this study, we investigated the impact of phytochemicals on reducing the transfer of an IncI1_Alpha plasmid carrying the *bla*_TEM-1B_ gene within poultry-related environmental settings. Previous studies have highlighted the presence of *β*-lactamase in Enterobacteriaceae from both human and poultry sources (57, 58), raising concerns due to its contribution to antimicrobial resistance in both animals and humans (59, 60). The donor organism in this study, *S.* Heidelberg, is one of the leading causes of foodborne outbreaks and often carries multiple drug-resistant genes. Our selection of *E. coli* as the recipient organism was based on its status as a common commensal bacterium present in both humans and animals. We examined horizontal gene transfer dynamics across different matrices, revealing variations in HGT frequencies and transconjugant counts, with chicken manure and ceca showing the highest transconjugant counts and conjugation frequencies, while water showed lower counts and frequency. Conjugation between fecal Enterobacteriaceae in water ecosystems was notably reduced due to nutrient deficiency, as observed in a previous study, which found that lower nutrient conditions significantly limited the transfer of antimicrobial resistance plasmids between microorganisms (61). The higher frequency of AR gene transfer could be attributed to the acquisition of the IncI1-I plasmid carrying *bla*_TEM-1B_ gene as confirmed by the WGS of the transconjugant *E. coli*. A previous WGS study also reported a high frequency of HGT via the acquisition of mobile genetic elements after experimental inoculation with the donor bacterium in gnotobiotic mice (86).

The disposal of chicken manure, typically through composting and anaerobic digestion, has been linked to the dissemination of AR bacteria and their ARGs into the farm environment and surrounding habitats. While these methods, along with chlorination of potable water, are partially successful in reducing AR bacterial populations across environments, they fall short in completely eliminating ARGs (62–64). The addition of nanoscale zero-valent iron to chicken manure compost reduces the concentrations of some antibiotics and decreases the abundances of antimicrobial resistance genes, with the reduction in the class 1 integron-integrase gene (intI1) playing a predominant role in HGT, offering a promising strategy to mitigate ARG release during composting (65). In this context, the effect of sub-inhibitory concentration of TC, CR, CA and BR on transconjugant counts and conjugation frequencies was investigated *in vitro* using various models, including broth, manure, and water. While CR and BR decreased conjugation frequency and transconjugant counts in broth and manure, CR and CA were effective in decreasing transconjugant count and HGT frequency in water. Earlier studies have demonstrated the inhibitory effects of various agents on bacterial conjugation, such as flavophospholipol (66), tanzawaic acids (67), synthetic 2-alkynoic fatty acids (68), non-specific chelating agents such as bisphosphonates (69), bile salts and bile acids (70), and *Carica papaya* seed macerate (71). A higher conjugation frequency of *bla*_TEM-1B_ was observed in manure than in water, which could be attributed to the higher organic content present in manure (72). All four tested phytochemicals effectively reduced the conjugative transfer frequency of *bla*_TEM-1B_ between *Salmonella* and *E. coli* (Tables 5–8), as evident from the significant reduction in transconjugant *E. coli* populations recovered on TSA plates containing ampicillin and nalidixic acid. These results suggest that a reduction of antimicrobial resistance propagation in the farm environment could potentially be achieved by phytochemical treatment of manure and water, however large-scale studies in a farm setting are warranted before recommending their application. Our study also explored the efficacy of TC and CR and their combinations as feed additives for reducing HGT frequency in a chicken gut model. Our findings indicate that in-feed supplementation of 0.5% TC effectively reduced HGT frequency in the ceca *ex vivo* on day 16 (Table 9). Acquisition of IncI plasmid from commensal *E. coli* was found to be responsible for the resistance phenotype demonstrated in experimentally inoculated *Salmonella* that was previously susceptible to antibiotics in chicken ceca (87).

Inhibition of HGT has been attributed to various aspects of the conjugation process. For example, phytochemical linalool was shown to reduce plasmid mobility (73), ZnCl_2_ downregulated the expression of conjugation-associated genes (74), and Fe_3_O_4_ composited with MoS_2_ reduced the cell membrane permeability against exogenous DNA (75). Similarly, free radicals reduced conjugation by attacking ARGs and disrupting cellular processes, including lowering reactive oxygen species levels, inhibiting DNA damage responses, impairing extracellular polymeric substance synthesis, and down-regulating plasmid transfer genes (76). However, the effects of phytochemicals on conjugation can vary depending on specific factors such as the type and concentration of phytochemicals, bacterial strains, and plasmids involved. Conjugation is dependent on the synthesis of the conjugative pilus, mediated by critical *tra* genes for pilus formation, extension, mating pair stabilization, and DNA transfer. Downregulation of these *tra* genes can inhibit pilus synthesis and suppress conjugation. Additionally, *finO* genes play a role in mating pair formation and DNA transfer, contributing to conjugation inhibition. Free nitrous acid treatment resulted in significant downregulation of genes responsible for DNA transfer and mating pair formation in *E. coli*, which was represented by a significant reduction in conjugation efficiencies (77). Likewise, gene expression studies conducted to determine the effect of hormone norepinephrine on conjugation frequency showed an upregulation in the majority of *tra* genes that coincided with an increased conjugation efficiency (78). Our RT-qPCR data revealed a significant downregulation in the majority of *tra* genes in treated *Salmonella* compared to the control. Moreover, there was a significant increase in the transcription of the fertility inhibition factor *fin*O when treated with BR, which decreases conjugation. Thus, the aforementioned RT-qPCR results suggest that the decreased HGT of *bla*_TEM_ from *Salmonella* to *E. coli* is mediated by their interference with pilus biogenesis. These results were supported by electron microscopy findings, which revealed reduced pilus formation on phytochemical-treated *Salmonella*.

Phytochemicals are biodegradable in soil and water (79), thus making them safe and environment-friendly compounds. Since the phytochemicals were used at their SIC against both *Salmonella* and *E. coli*, the attenuation of conjugation transfer observed was not due to bacterial growth inhibition but could be attributed to their potential effect on the plasmid bearing *bla*_TEM-1B_ or other conjugation mechanisms. Removal of the plasmid containing ARGs or plasmid curing could also result in reduced HGT frequency without impacting bacterial growth (80). However, results from the experiment determining the effect of phytochemicals on plasmid curing indicated no loss of *bla*_TEM-1B_ encoding plasmid from *Salmonella* cells. Collectively, our results suggest that phytochemicals exerted anti-HGT properties that could be attributed in part to the downregulation of genes, especially those associated with conjugation with a reduction in pilus formation.

## 5 Conclusion

To conclude, this study indicated that TC, CR, CA and BR could potentially be added to manure and water to reduce the transfer of antimicrobial resistance from *Salmonella* and *E. coli* in the farm environment. Since the animal gut is considered a potential environmental niche for HGT (10, 81), further investigation is warranted to ascertain the efficacy of these phytochemicals for reducing AR spread in the gut using chickens *in vivo*.

Several limitations should be considered when interpreting these findings. First, the study focused on a single plasmid type (IncI1-I[Alpha]) carrying *bla*_TEM-1B_ and one donor–recipient bacterial pair; therefore, the generalizability of the results to other plasmids, resistance genes, or bacterial species remains to be investigated. Second, experiments were conducted under controlled laboratory, simulated environmental, and *ex vivo* conditions, which may not fully capture the complexity of commercial poultry production systems, including microbial diversity, fluctuating environmental factors, and long-term ecological interactions.

Additionally, although reductions in HGT frequency were observed, the precise molecular pathways underlying gene regulation and pilus inhibition were not fully elucidated at the proteomic or structural level. Finally, while *ex vivo* results suggested reduced conjugation efficiency, *in vivo* efficacy, optimal dosing regimens, impacts on gut microbiota composition, animal performance, and economic feasibility were not investigated.

Future research should therefore include comprehensive *in vivo* trials, evaluation across diverse plasmid incompatibility groups and bacterial hosts, assessment of microbiome-wide effects, and mechanistic studies at transcriptomic and proteomic levels to validate and expand upon the anti-conjugative potential of these phytochemicals in real-world agricultural systems.

## Data Availability

The datasets presented in this study can be found in online repositories. The names of the repository/repositories and accession number(s) can be found below: https://www.ncbi.nlm.nih.gov/genbank/, PRJNA894991.

## Supplementary material

SUPPLEMENTARY FIGURE S1 Full-length, uncropped agarose gel image corresponding to Figure 1 showing colony PCR products from donor *Salmonella*, recipient *E. coli*, and transconjugant *E. coli*. Lanes include 100 bp DNA ladders at both ends and PCR amplicons for *bla*_TEM_, *invA*, and *uspA*. The regions presented in Figure 1 were derived from this original gel image. No lane rearrangement or selective editing was performed.
