# Supplementary material for: Evaluation of plant-based antimicrobials in controlling antimicrobial resistance gene spread in poultry production continuum
Source: Front Vet Sci. 2026 Jul 28;13:1837338. doi: 10.3389/fvets.2026.1837338 (PMC13459444; doi:10.3389/fvets.2026.1837338)
Supplement: Supplementary file 1 [file Table_1.docx]

Table S1. Composition of the Home Fresh Multi-Flock Chick N Game Starter/Grower used in this study.

| Component | Percentage |
| --- | --- |
| Crude Protein, min | 22.0% |
| Lysine, min | 1.25% |
| Methionine, min | 0.5% |
| Crude Fat, min | 3.5% |
| Crude Fibre, max | 4.0% |
| Calcium (Ca), min | 0.8% |
| Calcium (Ca), max | 1.3% |
| Phosphorous (P), min | 0.75% |
| Salt (NaCl), min | 0.25% |
| Salt (NaCl), max | 0.75% |
| Manganese (Mn), min | 135 ppm |
| Selenium (Se), min | 0.3 ppm |
| Vitamin A, min | 5,700 IU/lb |
| RVitamin E, min | 28 IU/lb |
| Phytase, min | 681 FYT^*^/lb |

*^*^One unit of phytase (FYT) is the amount of enzyme which liberated 1mmol of inorganic phosphate per minute from sodium phytate at pH 5.5 and 98.6^o^F.*
